# The European Badger (*Meles meles*) as an Indicator Host of Sylvatic *Trichinella britovi* Transmission in Western Romania

**DOI:** 10.3390/pathogens15060630

**Published:** 2026-06-12

**Authors:** Ana-Maria Marin, Dan-Cornel Popovici, Maria Monica Florina Moraru, Gianluca Marucci, Adriano Casulli, Francesco Celani, Sorin Morariu, Anamaria Plesko, Narcisa Mederle

**Affiliations:** 1 Department of Parasitology and Parasitic Diseases, Faculty of Veterinary Medicine, University of Life Sciences “King Mihai I”, 300645 Timisoara, Romania; anamaria.marin@usvt.ro (A.-M.M.);; 2 Department of Forestry, Faculty of Engineering and Applied Technologies, University of Life Sciences “King Mihai I”, 300645 Timisoara, Romania; 3 European Union Reference Laboratory for Parasites (EURL-P), Unit of Foodborne and Neglected Parasitic Diseases, Department of Infectious Diseases, Istituto Superiore di Sanità, 00161 Rome, Italyadriano.casulli@iss.it (A.C.);

**Keywords:** multiplex PCR, trichinellosis, western Romania, wildlife, zoonosis

## Abstract

Trichinellosis is an important parasitic zoonosis caused by nematodes of the genus *Trichinella*, affecting numerous carnivorous and omnivorous mammal species. In Europe, wildlife represents the main reservoir of *Trichinella* spp., contributing to the maintenance of the sylvatic cycle and acting as a potential source of infection for domestic animals and humans. In Romania, *Trichinella spiralis*, *Trichinella britovi*, and *Trichinella pseudospiralis* have been reported in domestic animals and wildlife, with *T. britovi* being frequently associated with the sylvatic cycle and wild carnivores. The aim of this study was to investigate the occurrence and molecular characterization of *Trichinella* larvae isolated from muscle tissues of European badgers (*Meles meles*) originating from different areas of Romania. Overall, muscle samples collected from 24 European badgers from six Romanian counties were examined using the artificial digestion method. Recovered larvae were subjected to PCR-based species discrimination by multiplex polymerase chain reaction (PCR). *Trichinella* larvae were detected in one of the 24 examined European badgers, originating from Arad County, Western Romania. Molecular analysis confirmed the presence of *T. britovi*, the species most commonly identified in wild carnivores from temperate regions. Although *T. britovi* has previously been reported in the European badger in Romania in a specimen from Central Romania, the present finding represents, to the best of our knowledge, the first detection of this parasite–host association in Western Romania. The low infection prevalence and the detection of *T. britovi* in the European badger indicate circulation within the sylvatic cycle and highlight the need for continued wildlife monitoring, given the zoonotic potential of this parasite.

## 1. Introduction

Mustelids (family Mustelidae) represent an important group of carnivorous mammals with a wide distribution in the Northern Hemisphere, as well as representatives in other geographical regions, adapted to diverse habitats ranging from forest ecosystems and agricultural areas to aquatic or peri-urban environments [1]. In the Romanian fauna, the family Mustelidae includes several species of ecological and epidemiological relevance, such as the European badger (*Meles meles*), European mink (*Mustela lutreola*), European pine marten (*Martes martes*), European polecat (*Mustela putorius*), Eurasian otter (*Lutra lutra*), least weasel (*Mustela nivalis*), stoat (*Mustela erminea*), and stone marten (*Martes foina*) [2]. Among these, the European badger is one of the most widely distributed mustelid species in Europe, occurring in a variety of habitats, including deciduous and mixed forests, grasslands, agricultural land, hilly areas, and regions located near human settlements.

European badger is the largest mustelid in Romania and exhibits predominantly nocturnal, terrestrial, and semifossorial behavior, living in complex burrow systems that are often used over long periods by family groups. Although it belongs to the order Carnivora, the European badger has an omnivorous and opportunistic diet, consuming invertebrates, small rodents, amphibians, reptiles, eggs, carrion, fruits, seeds, and other plant or animal resources available seasonally [3]. This flexible trophic behavior, associated with frequent movements between forests, agricultural land, and peripheral areas of settlements, may facilitate the European badger’s contact with various sources of pathogens, including nematodes with zoonotic potential.

Nematodes of the genus *Trichinella* are the etiological agents of trichinellosis, a zoonosis transmitted to humans through the consumption of raw or insufficiently cooked meat from animals infected with muscle larvae [4,5]. The infection may affect numerous species of carnivorous or omnivorous mammals, birds, and reptiles, and its distribution is considered nearly cosmopolitan, with the exception of Antarctica [5]. At present, the genus *Trichinella* includes several species and genotypes, divided into encapsulated and non-encapsulated forms. Among the encapsulated species, *T. spiralis* and *T. britovi* are of the greatest epidemiological importance in Europe, being frequently identified in both the domestic and sylvatic cycles [6,7].

In Europe, the sylvatic cycle of trichinellosis is maintained through complex ecological relationships, such as predation, scavenging, and the accidental or opportunistic consumption of infected tissues. Wild carnivores, particularly the red fox (*Vulpes vulpes*), the wolf (*Canis lupus*), the golden jackal (*Canis aureus*), the Eurasian lynx (*Lynx lynx*), the brown bear (*Ursus arctos*), and several mustelid species, are considered important hosts for the maintenance and spread of *Trichinella* spp. larvae in the natural environment [7,8]. Although the red foxes and the raccoon dog (*Nyctereutes procyonoides*) are often considered major reservoirs, European badger may play a relevant epidemiological role due to its omnivorous diet, its use of carrion as a food source, and its contact with habitats frequented by other carnivores and wild boars (*Sus scrofa*) [9].

In Romania, trichinellosis remains a zoonosis of major public health concern, with the main sources of human infection being meat from domestic pigs (*Sus scrofa domesticus*), wild boar, and, less frequently, other game species [10,11]. Studies conducted on wildlife in Romania have confirmed the circulation of *T. spiralis*, *T. pseudospiralis*, and *T. britovi*, the latter being frequently associated with the sylvatic cycle and identified in several wild carnivores and omnivores, including red foxes, wolves, golden jackals, Eurasian lynxes, wildcats (*Felis silvestris*), brown bears, wild boars, and some mustelids [10,12,13,14,15,16,17,18,19]. In this context, the European badger may represent a bridging host between forest ecosystems and rural environments, thereby indirectly contributing to the maintenance of the parasite in natural food webs.

The importance of European badger in the epidemiology of *Trichinella* spp. has been highlighted in several European countries, with available data supporting the role of mustelids as susceptible hosts and possible indicators of parasite circulation in sylvatic ecosystems. This species has been reported as a host for several *Trichinella* spp., mainly *T. britovi*, but also *T. spiralis* and *T. nativa*, depending on the geographical region and epidemiological context [20,21,22,23,24,25,26].

In Romania, to date, there has been only one report of *T. britovi* infection in European badger, representing an important contribution to understanding the diversity of hosts involved in the sylvatic cycle of trichinellosis [13]. However, data regarding the frequency of infection, geographical distribution, and actual role of this species in parasite transmission remain limited.

In this context, the aim of the present study was to investigate the presence and circulating species of *Trichinella* spp. in European badger specimens collected from different counties of Romania.

## 2. Materials and Methods

### 2.1. Sample Collection

**Figure 1:**
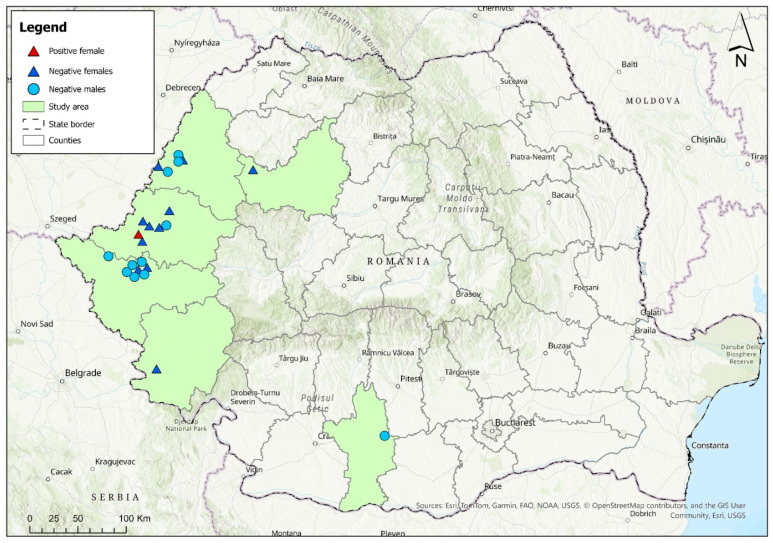
Geographical distribution of *Meles meles* specimen collected in the 2022–2025 period.

The study was conducted over a 3-year period, from 2022 to 2025, on a total of 24 European badgers, including 13 females and 11 males, collected from six counties in Romania (Figure 1). The animals were either found dead due to road traffic accidents or were legally hunted, in accordance with the annual quotas established by the Ministry of Environment, Waters and Forests. Hunting activities were carried out in compliance with Law No. 407/2006 on hunting and the protection of the hunting fund, as well as with national regulations on wildlife management [27]. The collected badgers were examined at the Parasitic Diseases Clinic of the Faculty of Veterinary Medicine Timișoara, University of Life Sciences “King Mihai I” from Timișoara.

Ethical approval was not required for this study, as no animals were captured, handled, euthanized, or sacrificed specifically for research purposes. Biological samples were obtained exclusively from *M. meles* found dead due to road traffic accidents or legally hunted during official hunting seasons, in accordance with national wildlife management regulations. Therefore, the study was based on opportunistic sampling and did not involve experimental procedures on live animals.

### 2.2. Diagnostic Procedures

Muscle samples were collected from each European badger and examined for the presence of *Trichinella* spp. larvae. Approximately 30 g of muscle tissue, mainly from the diaphragm and forelimb muscles, was collected from each animal. The samples were tested using the artificial digestion method, according to ISO 18743:2015/Amd 1:2023 lation (EU) 2015/1375 [28]. After digestion, the sediment was examined under a stereomicroscope for the detection of *Trichinella* spp. larvae. The recovered larvae were collected, counted to estimate the larval burden, and preserved in 96% ethanol until molecular analyses were performed.

Subsequently, the larvae were sent to the European Union Reference Laboratory for Parasites (EURL-P) in Rome, Italy, for species identification by multiplex PCR [29]. Briefly, DNA was extracted from individual larvae using the DNA IQ System kit and the Tissue and Hair Extraction kit, both manufactured by Promega (Madison, WI, USA). Five primer sets were used in the multiplex PCR, targeting specific regions of ribosomal DNA repeats, namely expansion segment V, ITS1, and ITS2, to obtain a species-specific electrophoretic profile of DNA bands [30,31]. Multiplex PCR products were analyzed by capillary electrophoresis using the QIAxcel Advanced system and a DNA High Resolution Cartridge. Electrophoretic separation was performed using the OM500 method managed by QIAxcel ScreenGel software (v. 2.1). The QX Alignment Marker 15–500 bp and the QX DNA Size Marker 25–500 bp were utilized for fragment analysis. Capillary electrophoresis reagents and instrumentation were from Qiagen GmbH (Hilden, Germany).

## 3. Results

**Figure 2:**
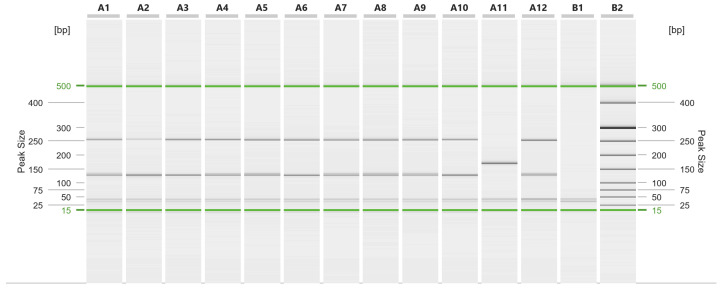
Capillary electrophoresis of multiplex PCR products obtained from *Trichinella* larvae collected from European badger. A1–A10: larvae isolated from European badger; A11: positive control for *Trichinella spiralis*; A12: positive control for *Trichinella britovi*; B1: negative control; B2: size marker.

Of the 24 European badger specimens examined, one (4.2%; 95% confidence interval, CI: 0.7–20.2) tested positive for *Trichinella* spp. by artificial digestion. The infected animal originated from Arad County, western Romania, at 46.22850° N latitude and 21.37390° E longitude. The larval burden was estimated at 0.9 larvae per gram (LPG) of muscle tissue, indicating a low-intensity infection. Ten *Trichinella* larvae were individually analyzed by multiplex PCR, and PCR-based fragment analysis confirmed the presence of *T. britovi*. Capillary electrophoresis of the multiplex PCR products obtained from the larval isolates revealed two bands of approximately 127 and 253 bp, respectively, consistent with *T. britovi* (Figure 2).

## 4. Discussion

When comparing the results of the present study with those reported in the literature, to the best of our knowledge, this represents the first detection and PCR-based species discrimination of *T. britovi* in European badger in western Romania. Although this parasite–host association has previously been reported in Romania in a European badger specimen originating from the central part of the country, the present finding extends the known geographical distribution of *T. britovi* in this host and confirms the contribution of European badger in the sylvatic cycle of *Trichinella* spp. infection.

*Trichinella britovi* is one of the main species involved in the sylvatic cycle of trichinellosis, with wild animals playing an essential role in the maintenance, amplification, and spread of the parasite in the natural environment, particularly through trophic relationships based on predation, scavenging, and the consumption of infected tissues [7]. Considered the most widespread species in wild carnivores in Europe, *T. britovi* has also been reported in wildlife from Asia, North Africa, and West Africa; moreover, it can infect domestic pig populations and is regarded as the second most important *Trichinella* sp. from a public health perspective [32,33]. In Europe, this species has been identified in numerous wild hosts, such as brown bears and Eurasian lynxes [7], wolves [7,34], red foxes [7], jackals [7,35], wildcats [7,36], raccoon dogs [7,18,37], European pine martens [16], Eurasian otters [7], wild boars [7,38], and Eurasian beaver (*Castor fiber*) [39]. However, information regarding the involvement of European badger in the sylvatic cycle of this species remains limited.

European badger is a host of epidemiological interest because, although it belongs to the order Carnivora, it has an omnivorous and opportunistic diet [40]. The consumption of invertebrates, small vertebrates, eggs, animal remains, and occasionally carrion may facilitate exposure to *Trichinella* spp. larvae, given that the sylvatic cycle of the parasite is maintained through trophic relationships such as predation, scavenging, and the consumption of infected tissues [7]. In addition, the use of various habitats, ranging from forests and agricultural land to rural or peri-urban areas, may facilitate indirect contact with other infected sylvatic hosts [40].

In Europe, *Trichinella* spp. infection in European badger has been reported sporadically but repeatedly in several regions, including Latvia [21], Estonia [22,23], Finland [41], Poland [25], Serbia [24], Bosnia and Herzegovina [26], Croatia [20], Italy [42], and Spain [43], with reported prevalence values ranging from 1.8% to 100%, depending on the country and the size of the examined samples. These data confirm that European badger can act as a host for species of the genus *Trichinella* circulating in Europe, including *T. britovi*, *T. nativa*, *T. pseudospiralis*, and *T. spiralis*. Outside Europe, *Trichinella* spp. infection in European badger has been reported in Kazakhstan, where Akibekov et al. [44] reported a prevalence of 66.7%, and the species identified was *T. nativa*.

In Romania, *Trichinella* spp. infection is considered widely distributed in wildlife, and *T. britovi* represents one of the main species associated with the sylvatic cycle. Previous studies have reported this species in several wild carnivores and omnivores, such as foxes, wolves, golden jackals, Eurasian lynxes, wildcats, brown bears, wild boars, and different mustelid species [10,12,13,14,15,16,17,18,19]. The first report of *T. britovi* in European badger in Romania was published by Boros et al. [13], who reported *Trichinella* spp. infection in this host with a prevalence of 1.64% and identified the parasite in a single specimen originating from the central part of the country. Similar to that report, in the present study the infection was detected in a single European badger from western Romania, with a larval burden of 0.9 LPG, close to the previously reported value of 0.7 LPG [13]. This suggests that European badger may act more as an occasional or secondary host than as a major reservoir. Nevertheless, the repeated identification of *T. britovi* in European badger from different regions of Romania indicates that badgers should not be excluded from epidemiological studies on *Trichinella* spp. infection in sylvatic environments.

Although European badger is not a common source of meat for human consumption in Romania, the presence of *T. britovi* in this host has indirect public health relevance. The detection of the parasite in European badger in Arad County indicates the existence of an active sylvatic cycle, which may involve other wild hosts as well as game species, particularly wild boar. Through predation, scavenging, or the consumption of infected animal remains, these species may maintain parasite circulation within local food webs. Therefore, monitoring European badger may complement the surveillance of other wild hosts and help identify areas where *Trichinella* spp. are actively circulating in wildlife.

Human infection risk is mainly associated with the occurrence of *Trichinella* spp. infected animals that may enter the food chain, particularly wild boars, rather than with the mere presence of a specific host species in the environment. In this context, the consumption of raw or insufficiently cooked wild boar meat may represent a potential source of human infection, even when the larval burden is low [45]. In several European countries, human trichinellosis cases caused by *T. britovi* and associated with the consumption of infected wild boar meat have been reported in Greece [46], Serbia [47], Slovakia [48], Italy [49], France [50], Spain and Sweden [51,52], and Portugal [53]. In Romania, available data indicate that human trichinellosis has so far been associated with *T. spiralis*, with no confirmed reports of cases attributed to *T. britovi* [54].

Considering the limitations of the present study, namely the examination of a small number of European badgers and the identification of *T. britovi* infection in a single specimen with low parasite intensity, the results do not suggest that this species plays a major role as a reservoir host, although they do confirm its exposure to the parasite. European badger may acquire the infection through scavenging on infected carcasses and may subsequently contribute, albeit to a limited extent, to the maintenance of *Trichinella* spp. in the sylvatic environment.

## 5. Conclusions

The present study reports the first detection and PCR-based species discrimination of *Trichinella britovi* in European badger in western Romania, extending the known distribution of this host–parasite association and confirming the active circulation of the parasite within the sylvatic cycle. Given the zoonotic nature of *T. britovi*, the results support the need for an integrated One Health approach including wildlife surveillance, veterinary control of game meat, and continuous monitoring of public health risk.

## Data Availability

The original contributions presented in this study are included in the article. Further inquiries can be directed to the corresponding author.
